# NADPH oxidase is implicated in the pathogenesis of oxidative phosphorylation dysfunction in mice fed a high-fat diet

**DOI:** 10.1038/srep23664

**Published:** 2016-05-13

**Authors:** Inmaculada García-Ruiz, Pablo Solís-Muñoz, Daniel Fernández-Moreira, Montserrat Grau, Teresa Muñoz-Yagüe, José A. Solís-Herruzo

**Affiliations:** 1 Centro de Investigación, Laboratorio de Gastroenterología y Hepatología, Hospital Universitario 12 de Octubre, Universidad Complutense, 28041-Madrid, Spain; 2 Institute of Liver Studies, King’s College Hospital, SE5 9RS, London, United Kingdom; 3 Servicio de Bromatología e Higiene Alimentaria, Centro Militar de Veterinaria del Ministerio de Defensa, 28024-Madrid, Spain

## Abstract

The aim of this study was to evaluate the role of NADPH oxidase (NADPHox) in the pathogenesis of oxidative phosphorylation (OXPHOS) dysfunction as found in mice fed a high-fat diet (HFD). C57BL/6J mice were distributed in four groups: WT/SCD: six wild-type (WT) mice fed a standard chow diet (SCD); WT/HFD, six WT mice fed a HFD; NOX2^−/−^/SCD, six NADPHox-deficient mice on a SCD; (4) NOX2^−/−^/HFD, six NADPHox-deficient mice on a HFD. After 32 weeks, we studied the liver for: histology; OXPHOS complex activity; fully assembled OXPHOS complexes and their subunits; gene expression of OXPHOS subunits; oxidative and nitrosative stress; and oxidative DNA damage. In the liver of WT/HFD mice, we found a significant decreased in the activity of all OXPHOS complexes, in fully assembled complexes, in the amount of OXPHOS subunits, and in gene expression of mitochondrial DNA-encoded subunits. 8-hydroxy-2′-deoxyguanosine was only increased in mitochondrial DNA. The liver of NOX^−/−^/HFD mice showed mild steatosis but no non-alcoholic steatohepatitis (NASH) lesions were found. OXPHOS activity, OXPHOS subunits, and assembly of subunits into OXPHOS complexes were normal in these mice. We conclude that this study shows that NADPH deficiency protects mice from developing OXPHOS dysfunction and NASH caused by a HFD.

Nonalcoholic fatty liver disease (NAFLD) is a clinico-pathological condition characterized by histological features of alcoholic liver disease in patients who do not consume significant amounts of alcohol. It includes a wide spectrum of liver diseases ranging from simple fatty liver to non-alcoholic steatohepatitis (NASH), which may progress to more severe liver complications such as cirrhosis and hepatocellular carcinoma^1^. NAFLD has become an important public health problem because of its high prevalence^2^, potential progression to severe liver disease, and strong link with important cardiometabolic risk factors^3^. Although the pathogenesis of NAFLD remains undefined, a so-called ‘two hits’ model has been proposed^4^. The ‘first hit’ is related to insulin resistance, which increases lipolysis, particularly of the visceral adipose tissue, and determines an accumulation of fat in the liver. The ‘second hit’ involves oxidative stress, resulting in inflammation, stellate cell activation, and fibrogenesis^5^. In previous studies, we showed that NAFLD lesions, including NASH lesions, can be prevented by treating *ob/ob* mice or mice on a high-fat diet (HFD) with antioxidants or antiperoxynitrites^6^,^7^,^8^, thus suggesting that nitro-oxidative stress may play a critical role in the pathogenesis of these lesions. The cause of this stress remains unclear. Potential sources of oxidative stress are multiple, including cytochrome P450-2E1 (CYP2E1)^9^, xanthine oxidase (XDH)^10^, mitochondrial electron transport chain^11^, and nicotinamide adenine dinucleotide phosphate-oxidase (NADPHox)^12^. CYP2E1, a member of the oxide reductase cytochrome family, may oxidize a variety of small molecules^13^ to produce superoxide anion, a very potent reactive oxygen species (ROS). Both the activity and expression of this enzyme is increased in the liver of patients and animals with NASH^9^,^14^ and this increase correlates with NAFLD severity. Likewise, XDH activity is significantly increased in mouse models of NAFLD and these lesions can be prevented by inhibiting XDH activity in these animals^15^. However, we showed that silencing XDH with appropriated small interfering RNAs did not prevent nitro-oxidative stress caused by saturated fatty acids in HepG2 cells^16^.

Mitochondria are one of the most important sources of ROS^17^. In previous studies, we showed that oxidative phosphorylation (OXPHOS) is defective in individuals with NASH^18^, in *ob/ob* mice with NAFLD^6^, and in mice on a HFD^7^. In these obese mice, we found evidence that OXPHOS inhibition was caused by a reduced amount of fully assembled complexes because of subunit decreased synthesis and increased degradation by nitro-oxidative stress.

NADPHox is a multiprotein complex found in all types of liver cells, including hepatocytes, which may cause oxidative stress by reducing molecular oxygen to superoxide and hydrogen peroxide^12^. The role played by NADPHox in the pathogenesis of NASH is not well known. De Minicis *et al*.^19^ showed that NADPHox-deficient mice are resistant to liver fibrosis not only in NASH, but also in other animal models of liver injury, which might be ascribed to the role played by NADPHox in the activation of hepatic stellate cells and the progression of fibrosis in NASH^20^,^21^. However, other authors have reported that steatosis development, lipid peroxidation, inflammation, and pericellular fibrosis caused by a methionine and choline-deficient (MCD) diet is independent of NADPHox^22^, as the same histological lesions were observed in the liver of NADPHox-deficient and wild-type mice fed a MCD diet. However, this animal model of NASH has been criticised for not replicating the phenotype and pathogenic mechanism of human NASH. Thus, mice on a MCD diet are cachectic, have low plasma levels of triglycerides, exhibit a different hepatic steatosis distribution, and are not insulin resistant^23^. In previous studies, we showed that both the gene expression of the molecular components of NADPHox and its enzyme activity are increased in the liver of mice fed a HFD^7^. A number of factors may induce NADPHox activity, including saturated fatty acids^16^, TNFα^24^, and TGFβ^25^, whose concentrations are increased in the liver of obese mice^6^. The oxidative stress caused by increased NADPHox activity might determine OXPHOS dysfunction, which in turn would give rise to a vicious cycle contributing to increase oxidative stress. Although our *in vitro* studies have provided evidence that NADPHox may be a major source of nitro-oxidative stress^16^, no evidence for this role has been identified *in vivo*. Therefore, the aims of this study were to determine whether increased NADPHox activity in the liver is implicated in the pathogenesis of the nitro-oxidative stress found in mice on a HFD and to understand the role played by this enzyme complex in the dysfunction of OXPHOS in these mice.

## Results

### NADPHox deficiency mitigated the effects of a HFD on the hepatic triglyceride, free fatty acid (FFA), and TNFα levels, as well as on the plasma levels of glucose, triglycerides, FFAs, adiponectin, and transaminases

As shown in Table 1, the body weight gain over the 32 weeks of the experiment was significantly higher in WT/HFD mice when compared to WT/SCD mice. This increase was associated with increased triglyceride, FFA, and TNFα levels in the liver. In addition to obesity, WT/HFD mice developed other features of the metabolic syndrome, including hyperglycemia, hypertriglyceridemia, increased plasma FFAs, and low levels of plasma adiponectin. Because NADPHox may play a major role in the pathogenesis of oxidative stress in NASH, we fed NADPHox-deficient mice a HFD for 32 weeks. Although the final body weight of these deficient mice was in the range of obesity^26^ their hepatic triglyceride, FFA, and TNFα concentrations were significantly lower than in WT/HFD mice. Likewise, serum aminotransferase, glucose, FFA, and triglyceride levels were significantly lower in NOX2^−/−^/HFD mice than in WT/HFD mice. In contrast, plasma adiponectin remained at control levels in NOX2^−/−^ mice on a HFD (Table 1).

### Non-alcoholic steatohepatitis induced by a HFD was mitigated in NADPHox -deficient mice

**Figure 1:**
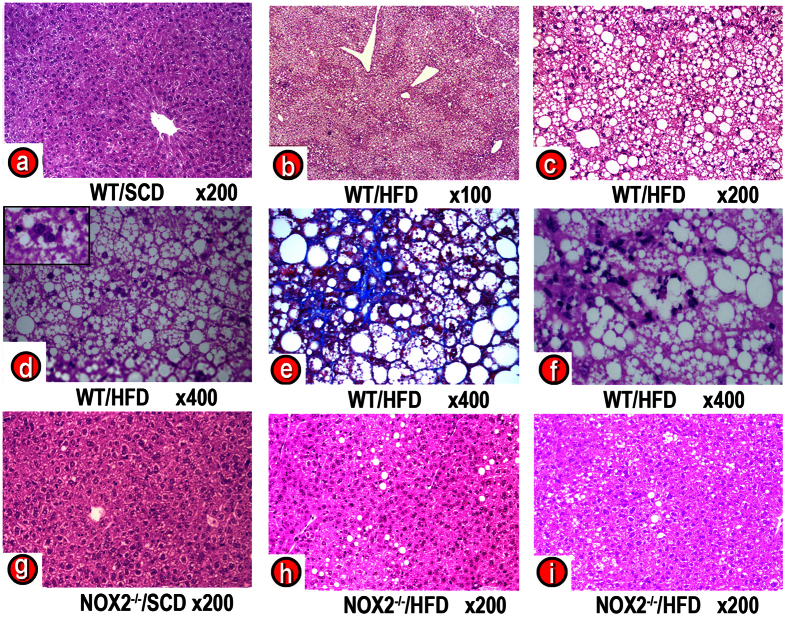
Effects of NADPH oxidase deficiency on liver histology of HFD-fed mice. Liver histology.(**a**) Wild-type mouse on a standard chow diet (WT/SCD). (**b**–**f**) Wild-type mouse on a high fat diet (WT/HFD). (**b,c,e**) Severe steatosis. (**d**) Hepatocyte with ballooning degeneration and Mallory body. (**e**) Perisinusoidal fibrosis. (**f**) Mixed neutrophilic-lymphocytic infiltrate. (**g**) NOX2^−/−^ mouse on a SCD (NOX2^−/−^/SCD). (**h,i**) NOX^−/−^ mouse on a HFD (NOX2^−/−^/HFD). Liver samples were stained with hematoxylin-eosin (**a–d,f–i**) or Masson’s-trichrome stain (**e**). Magnification 100x (**b**), 200x (**a,c,g,h, i**), 400x (**d,e,f**).

**Figure 2:**
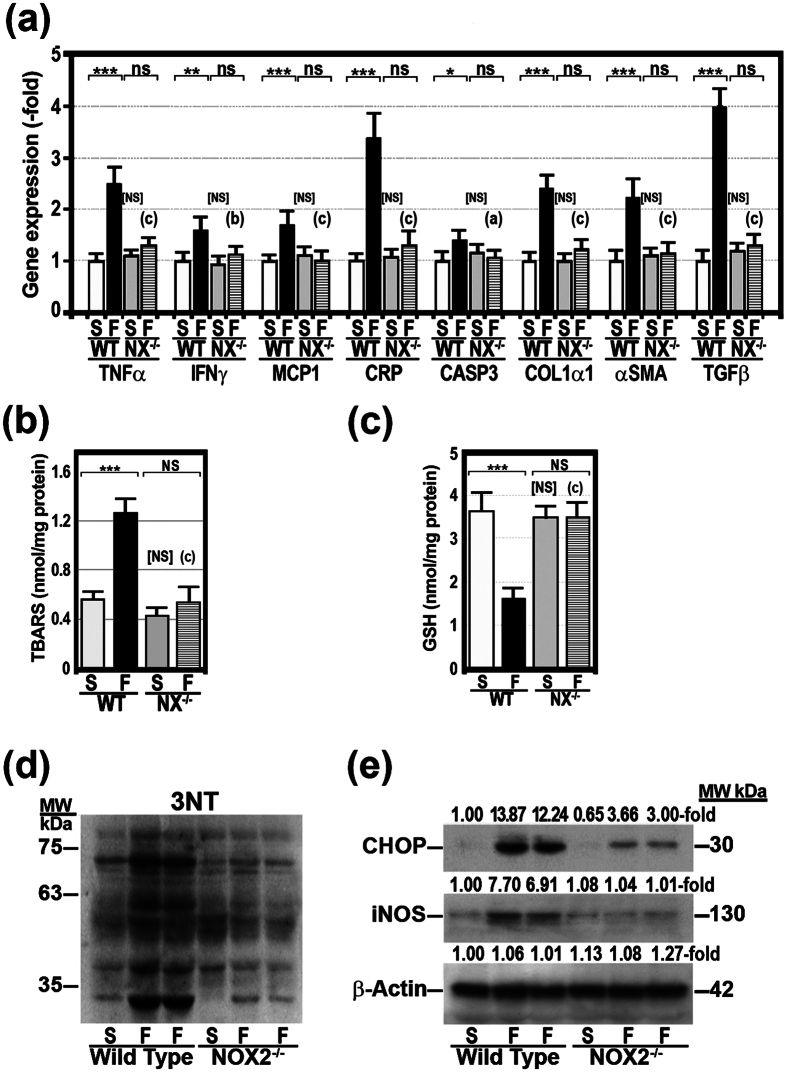
The effects of a HFD on inflammation, apoptosis, and fibrogenesis markers, as well as on nitro-oxidative and endoplasmic reticulum stress, were absent in NADPHox-deficient mice. (**a**) The gene expression of TNFα, IFNγ, MCP-1, CRP, caspase-3, collagen α1(I), αSMA, and TGFβ1 was measured by RT-PCR. WT, wild-type mice; NX^−/−^, NADPH oxidase-deficient mice; S, mice on a standard chow diet; F, mice on a high-fat diet. (**b**) Thiobarbituric acid reactive substances (TBARS) and (**c**) reduced glutathione (GSH) were measured in the liver as described under “Methods”. Results are expressed as fold over the control level. *p < 0.05; **p < 0.01; ***p < 0.001; ns, not significant as compared HFD-fed mice with SCD-fed mice. (**a**) p < 0.05; (**b**) p < 0.01; (**c**) p < 0.001 as compared WT/HFD mice with NOX2^−/−^/HFD mice. [NS], not significant as compared WT/SCD mice with NOX2^−/−^/SCD mice. (**d**) Liver proteins were analysed by Western blotting. Membranes were probed with specific antibody against 3-nitrotyrosine (3-NT). MW, molecular weight. (**e**) Liver proteins were isolated from wild-type and NADPH oxidase (NOX2^−/−^)-deficient mice fed a SCD or a HFD as indicated above and protein expression of was analysed by Western blotting. Membrane was probed with specific antibody against C/EBP homologous protein (CHOP), inducible nitric oxide synthase (iNOS), and β-actin.

As compared with WT/SCD mice, the liver of WT/HFD mice revealed severe steatosis, ballooning degeneration, Mallory bodies, scattered mixed neutrophilic-lymphocytic inflammatory foci, and increased perisinusoidal fibrosis (Fig. 1). These histological results were supported by the analysis of hepatic triglyceride levels (Table 1) and the gene expression of inflammatory markers [TNFα, monocyte chemoattractant protein-1 (MCP-1), IFNγ, and C-reactive protein (CRP)], apoptosis markers (caspase-3), and fibrogenesis markers [collagen α1(I), α–smooth muscle actin (αSMA) and TGFβ)], the levels of which were significantly increased in the liver of WT/HFD mice (Fig. 2a). In these livers, we also found evidence of oxidative, nitrosative and endoplasmic reticulum stress. Thus, WT/HFD mice had a marked increase in TBARS (Fig. 2b) and a decrease in reduced glutathione (GSH) (Fig. 2c). The latter decrease was not caused by a reduced synthesis of glutathione, as gene expression of nuclear factor erythroid 2-related factor 2 (NRF2) was not decreased. NRF2 is a transcription factor that regulates the expression of antioxidant factors, including glutathione, and protects against oxidative damage^27^, (Supplementary Fig. S1). Likewise, the expression of 3-tyrosine-nitrated proteins (Fig. 2d and Supplementary Fig. S2), inducible nitric oxide synthase (iNOS) and C/EBP homologous protein (CHOP) (Fig. 2e), a marker of endoplasmic reticulum (ER) stress^28^, was strikingly increased in these mice. All these markers were normal or markedly reduced in NADPHox-deficient mice on a HFD (Fig. 2a). Only CHOP expression remained slightly elevated in NOX2^−/−^/HFD mice despite the absence of oxidative stress. The liver histology of NOX2^−/−^/HFD mice showed mild steatosis but no ballooning degeneration, Mallory bodies, infiltrates, or fibrosis (Fig. 1).

### Activity of OXPHOS enzyme complexes, ATP content and ATP/ADP ratio were normal in the liver of NADPHox-deficient mice on a HFD

**Figure 3:**
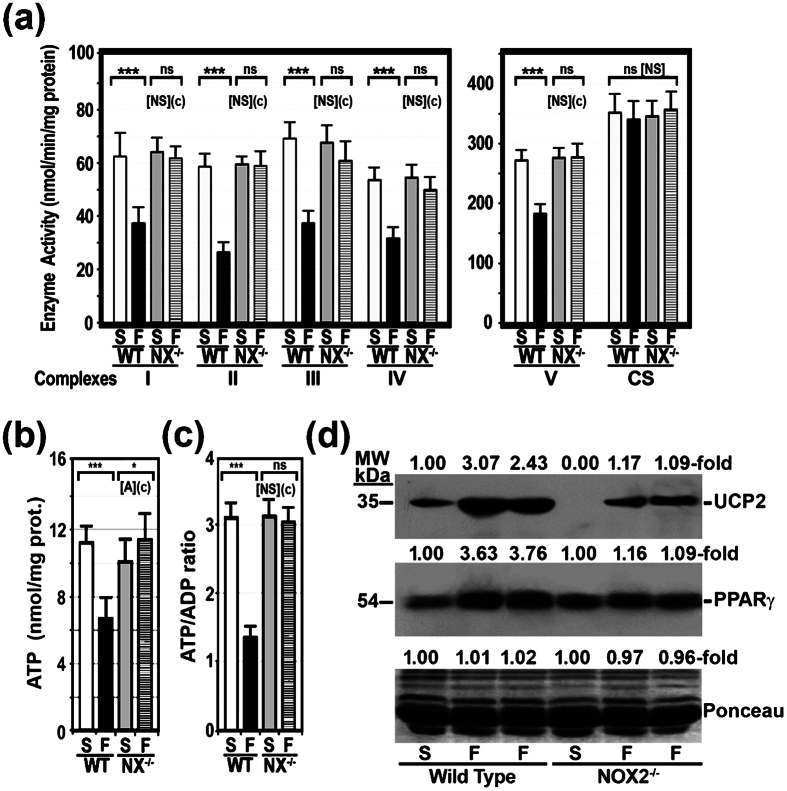
High-fat diet did not alter OXPHOS complex activity in NADPHox-deficient mice. (**a)** The activity of OXPHOS enzyme complexes and citrate synthase (CS) was measured as indicated in “Methods”, and expressed as nmol of substrate used per minute per mg of protein; it is also referred to as a percentage of the specific activity of citrate synthase (CS). **(b)** ATP content. **(c)** ATP/ADP ratio in the liver of mice treated as indicated. Values are shown as mean ± SD. (**c**), p < 0.001 comparing WT/HFD mice with NOX2^−/−^/HFD mice. ns, not significant; ***p < 0.001 as compared HFD-fed mice with SCD-fed mice. [NS], not significant; [A], p < 0.05 as compared WT/SCD mice with NOX2^−/−^/SCD mice. WT, wild-type mice; NX^−/−^, NADPH oxidase-deficient mice; S, mice fed a standard chow diet; F, mice fed a high-fat diet. **(d)** Western blot showing hepatic protein expression of UCP-2, PPARγ, and Ponceau staining in the same groups of mice as above. MW, molecular weight.

We measured the enzymatic activity of OXPHOS complexes in the liver of WT and NADPHox-deficient mice fed a SCD or a HFD. To correct for mitochondrial volume, all complex enzyme activities were normalized to the activity of citrate synthase (CS). The enzyme activity of the five OXPHOS complexes was reduced to 59.8 ± 9.9%, 45.2 ± 8.4%, 54.1 ± 9.6%, 59.2 ± 5.6%, and 67.2 ± 15.2%, respectively, in obese WT/HFD mice as compared to control WT/SCD mice. In contrast, the activity of all these OXPHOS complexes was similar in NOX2^−/−^/HFD mice and NOX2^−/−^/SCD mice (Fig. 3a).

This defect in OXPHOS activity was not limited to the liver of WT/HFD mice, since we also identified it in the visceral fat of these obese mice. As in the case of the liver, the activity of OXPHOS complexes was normal in the visceral fat of NOX2^−/−^/HFD mice (Supplementary Fig. S3).

We also measured ATP content and ATP/ADP ratio in the liver of the four groups of mice. As shown in Fig. 3b, HFD decreased hepatic ATP from 11.3 ± 0.4 nmol/mg protein in WT/SCD mice to 6.7 ± 1.1 nmol/mg protein (p < 0.001) in WT/HFD mice. However, this diet did not decrease ATP content in the liver of NOX2^−/−^/HFD mice. Likewise, the ATP/ADP ratio was significantly decreased in WT mice on a HFD, but remained unchanged in NOX2^−/−^ mice on the same diet (Fig. 3c).

Finally, uncoupling protein-2 (UCP2) and peroxisome proliferator-activated receptor gamma (PPARγ), a transcription factor that upregulates *Ucp-2* gene expression, were markedly increased in WT mice fed a HFD. This increase was not found in NOX^−/−^/HFD mice (Fig. 3d). Likewise, silencing *NOX2* in HepG2 cells prevented the increase in UCP2 and PPARγ protein levels caused by treating cells with 200 μM palmitic or stearic acids. (Supplementary Fig. S4).

### Fully assembled OXPHOS complexes were decreased in the liver of HFD-fed mice but not in NADPHox-deficient mice fed the same diet

**Figure 4:**
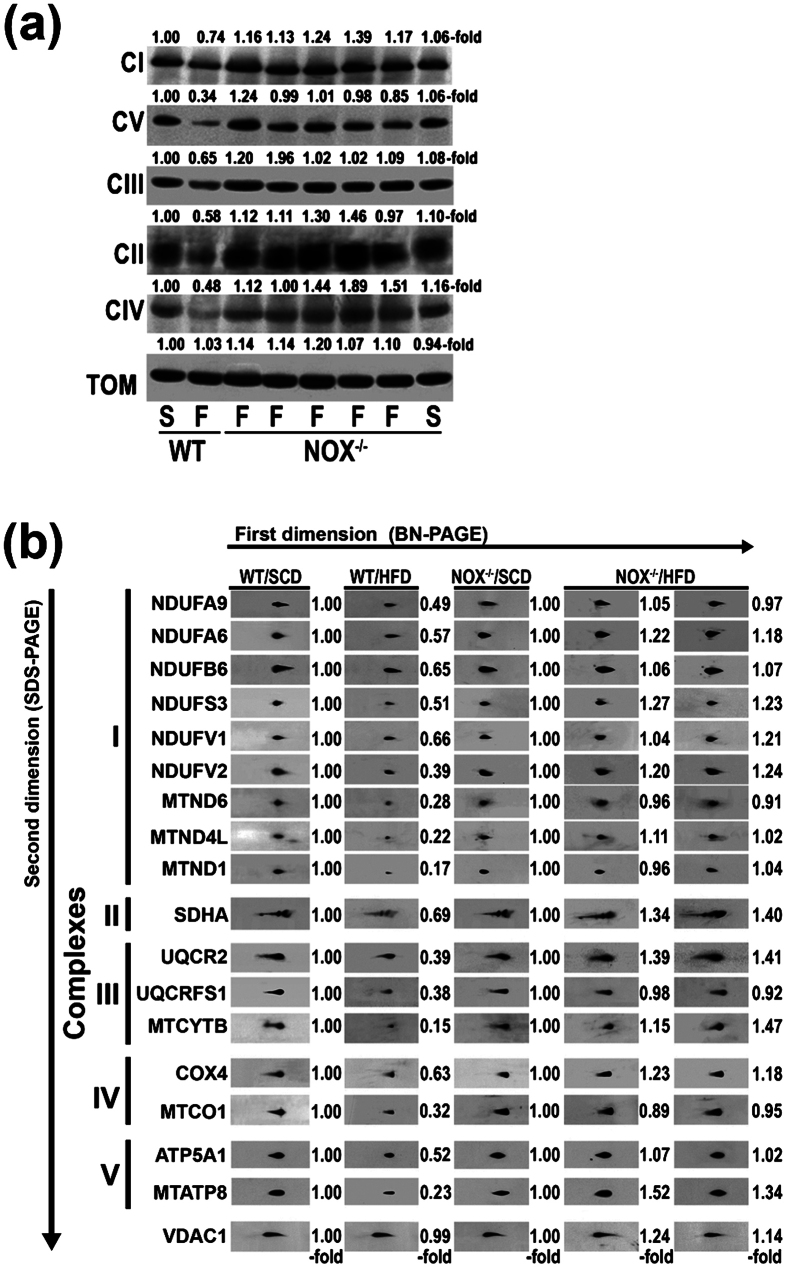
NADPHox-deficient mice were protected against the effects of a high-fat diet on the amount of complex subunits and assembly of OXPHOS complexes. (**a**) BN-PAGE analysis of OXPHOS complexes in WT and NADPHox-deficient (NOX^−/−^) mice fed a standard chow (S) or a high-fat (F) diet. A Western blot analysis of mitochondrial proteins was performed using antibody against complex I subunit NDUFA9, complex II subunit SDHA, complex III subunit UQCR2 protein, complex IV subunit MTCO1, complex V subunit ATP5A1, and TOM complex subunit TOM20. The expression of TOM complex (TOM) was used as loading control. (**b**) The mitochondrial proteins extracted from the same groups of mice were separated in the first dimension using BN-PAGE, and then in the second dimension using SDS-PAGE. The presence of individual subunits of these complexes was identified by immunoblotting using appropriated antibodies. The expression of VDAC1 was used as loading control. X-fold, amount of subunit in HFD-fed mice (WT or NOX^−/−^) divided by amount of same subunit in control mice, whether WT/SCD or NOX^−/−^/SCD.

The first-dimension BN-PAGE system illustrates that the abundance of fully assembled complexes was markedly diminished in wild-type mice fed a HFD as compared with WT/SCD mice (Fig. 4a), which concurs with the decreased OXPHOS-complex activity found in these obese mice. However, NADPHox-deficient mice on a HFD (NOX^−/−^/HFD) for 32 weeks exhibited normal or even increased levels of fully assembled complexes in mitochondrial preparations (Fig. 4a).

To study how mitochondrial complex subunits were affected by HFD, complexes were resolved by second-dimension SDS-PAGE and nDNA- and mtDNA-encoded subunits were detected using specific antibodies. As Fig. 4b shows, the most striking finding was a fall in the amount of all studied OXPHOS subunits in WT/HFD mice. This reduction was particularly marked for mtDNA-encoded subunits. Thus, while the amount of nDNA-encoded subunits was decreased to 53.2 ± 12.2% of control values in WT/HFD mice, mtDNA-encoded subunits were reduced to only 23.6 ± 6.4% of the amount found in control mice (p < 0.0001). In NADPH-deficient mice fed the same diet, the protein content of all studied subunits was normal or over the control levels (Fig. 3b).

### HFD did not decrease mitochondrial DNA (mtDNA)-encoded OXPHOS subunits in NADPHox-deficient mice because mtDNA is protected against the oxidative damage caused by HFD in these mice

**Figure 5:**
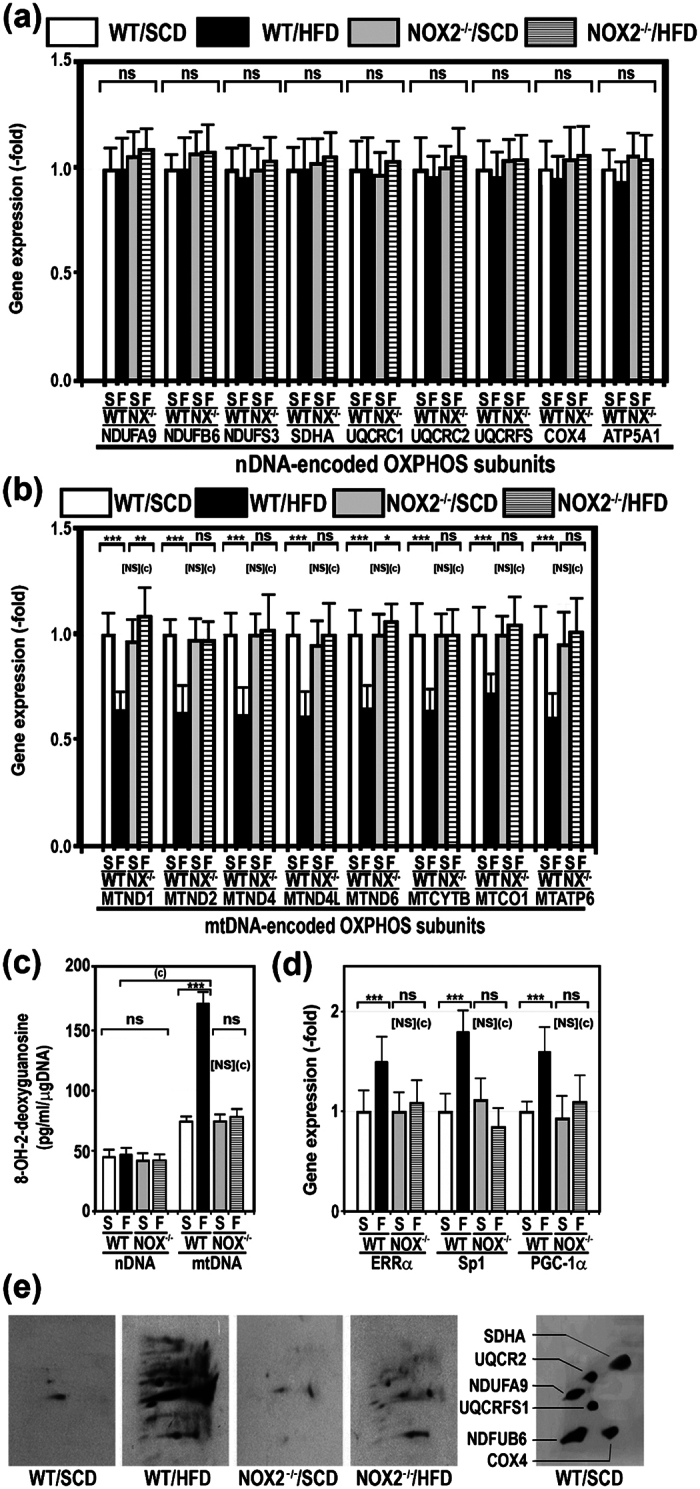
HFD did not suppress the gene expression of mtDNA-encoded OXPHOS subunits in NADPHox-deficient mice. (**a,b)** The gene expression of OXPHOS subunits was analysed by quantitative real-time PCR in mice treated as described in Fig. 1. Gene expression was expressed as subunit/β-actin mRNA ratio. **(c)** 8-Hydroxy-2′-deoxyguanosine content was measured in the nDNA and mtDNA of these same groups of mice. **(d)** Effects of a HFD on oestrogen-related receptor α (ERRα), specific protein 1 (Sp1) and peroxisome-proliferator activated receptor (PPARγ) coactivator-1α (PGC-1α) gene expression in WT and NOX2^−/−^ mice. The messenger RNAs for ERRα, Sp1, and PGC1α were analysed in the liver of mice by RT-PCR following the procedure described in the “Methods” section. The factor/β-actin mRNA ratio was calculated. Data are expressed as fold change over SCD-fed mice. *p < 0.05; **p < 0.01; ***p < 0.001; ns, not significant as compared HFD-fed mice with SCD-fed mice. (**c**), p < 0.001 as compared WT/HFD mice with NOX2^−/−^/HFD mice. [NS], not significant as compared WT/SCD mice with NOX2^−/−^/SCD mice. (**e**) Mitochondrial proteins extracted from the four groups of mice were separated in the first dimension using BN-PAGE and in the second dimension using SDS-PAGE. The presence of 3-tyrosine-nitrated proteins was identified by immunoblotting using a specific antibody against 3-nitrotyrosine. The membrane of a WT/SCD mouse was probed with antibody against OXPHOS subunits NDUFA9, NDUFB6, UQCR2, UQCRFS1, COX4, and SDHA.

To determine whether gene expression of OXPHOS complex subunits was decreased in NADPHox-deficient mice on a HFD, we examined the steady-state levels of some representative nDNA- and mtDNA-encoded mRNAs in the liver of all four groups of mice. This study revealed that the gene expression of nDNA-encoded subunits was not decreased in any group of mice regardless of the diet they were fed (Fig. 5a). On the contrary, the gene expression of mtDNA-encoded OXPHOS subunits was reduced to 62.4 ± 7.1% in WT/HFD mice. This decrease was not observed in NADPHox-deficient mice on a HFD (Fig. 5b).

The 8-hydroxy-2′-deoxyguanosine (8-OHdG) content in nDNA was identical in all groups of mice (Fig. 5c). However, when compared with the content of 8-OHdG in nDNA, this marker of oxidative DNA damage was significantly increased in the mtDNA isolated from all groups of mice, most particularly in the mtDNA from WT mice on a HFD (Fig. 5c). The levels of 8-OHdG in mtDNA did not increase in NADPHox-deficient mice despite their being fed a HFD.

### HFD upregulated the gene expression of oestrogen-related receptor α (ERRα), specific protein 1 (Sp1), and PPARγ coactivator 1α (PGC-1α) in wild-type mice but not in NADPHox-deficient mice

To determine whether HFD modifies the gene expression of transcription factors involved in the upregulation of genes encoding OXPHOS proteins, we measured the gene expression of ERRα, Sp1, and PGC-1α by RT-PCR. As shown in Fig. 5d, HFD significantly increased gene expression for these three transcription factors in wild-type mice but not in NADPHox-deficient mice.

Since 3-tyrosine nitration may accelerate mitochondrial protein degradation^29^,^30^, we analysed the presence of 3-tyrosine nitrated mitochondrial proteins in the liver of all four groups of mice. As shown in Fig. 5e, the mitochondrial proteins of WT/HFD mice were intensely nitrated at 3-tyrosine residues, while this nitration was less intense in the mitochondrial proteins of NADPHox-deficient mice fed the same diet.

## Discussion

In previous studies, we showed that the gene expression and enzymatic activity of the NADPHox complex are increased in mice on a HFD and we suggested that this enzyme system may play a role in the pathogenesis of oxidative stress in NASH and in the reduction of OXPHOS activity in these obese mice^7^. Moreover, we have already shown that the saturated fatty acids contained in HFD may upregulate the gene expression of NADPHox components and increase its enzymatic oxidative activity in HepG2 cells^16^. These acids also reduced the amount of OXPHOS subunits, fully assembled complexes and hence OXPHOS activity^16^. The reduced amount of OXPHOS subunits and fully assembled complexes was due to decreased gene expression for mtDNA-encoded subunits and the accelerated degradation of all OXPHOS subunits. These effects of saturated fatty acids are primary mediated by NADPHox, since they did not occur in HepG2 cells with silenced Rac1, a component of the NADPHox complex^16^. In the present study, we confirm that this oxidative enzyme complex, whose activity was increased in WT/HFD mice (Supplementary Fig. S5), plays a role in the pathogenesis of NASH and OXPHOS dysfunction caused by HFD. Thus, we have found that NADPHox-deficient mice on a HFD show lower plasma glucose, triglyceride and free fatty acid levels when compared to wild-type mice on the same diet, even though this diet caused a similar weight gain in both NADPHox-deficient mice and wild-type mice. Moreover, plasma adiponectin remained at control levels in NADPHox-deficient mice on a HFD. Although the liver of these mice showed steatosis, this was markedly less intense than in WT/HFD mice, and was not associated with steatohepatitis lesions, such as ballooning degeneration, Mallory bodies, inflammatory infiltrates, or fibrosis (Fig. 1). In agreement with these histological findings, the study of the liver tissue showed that hepatic triglycerides, although increased, were significantly lower in NOX2^−/−^/HFD mice when compared to WT/HFD mice (Table 1). Likewise, both hepatic FFA concentrations and gene expression for inflammation, apoptosis, and fibrogenesis markers were significantly lower in NOX2^−/−^/HFD mice than in WT/HFD mice (Fig. 2a). Moreover, oxidative stress markers (TBARS, GSH) and nitrosative stress markers (iNOS, 3-tyrosine nitrated proteins) were similar in NOX2^−/−^/HFD mice and in WT/SCD as well as in NOX2^−/−^/SCD mice (Fig. 2 and Supplementary Fig. S2). This protective effect of NADPHox deficiency was not secondary to reduced caloric intake or lower final body weight, since both features were similar in WT/HFD and NOX^−/−^/HFD mice (Table 1). Similarly, the final weight of NOX^−/−^/HFD mice was in the range of obese mice.

The HFD induced ER stress as the CHOP expression was markedly elevated in WT/HFD mice. This stress was reduced, although present, in NOX^−/−^/HFD mice despite the absence of oxidative stress. This increase may be ascribed to the fact that fatty acids can also cause ER stress through a direct mechanism. These acids are rapidly incorporated into phospholipids in the rough ER leading to changes in ER structure and to ER stress^31^.

An interesting finding in NADPHox-deficient mice on a HFD was their markedly decreased steatosis grade. This is a phenomenon that we had already observed in *ob/ob* mice treated with antioxidants or antiperoxynitrite^6^,^8^,^29^. In these obese animals, steatosis was absent or very mild. This histological finding may also result from the lesser nitro-oxidative stress seen in NADPHox-deficient mice. Thus, the *in vitro* exposure of hepatic proteins to peroxynitrite reduces the amount of ApoB100 and ApoB45^8^, two proteins involved in both the assembly of triglycerides into very-low-density lipoproteins (VLDL) and VLDL secretion^32^. This effect of peroxynitrite was prevented in the presence of melatonin^8^. Other authors have also shown that oxidative stress may disrupt the structure of ApoB100 and reduce its secretion by HepG2 cells^33^.

In previous studies, we showed that the activity of OXPHOS complexes was significantly decreased in patients with NASH^18^, in *ob/ob* mice^6^, and in mice on a HFD^7^. As mentioned above, this mitochondrial dysfunction is caused by a reduced synthesis of mtDNA-encoded OXPHOS subunits and probably by an accelerated degradation of all OXPHOS subunits^7^,^16^. These defects must also be due to the nitro-oxidative stress present in the liver of these obese mice. A number of factors may contribute to generate this stress including CYP2E1, XDH, NADPHox, and a defective OXPHOS system, among others. In the present study, we show that HFD induced a marked and significant decrease in the enzymatic activity of OXPHOS complexes in wild-type mice but not in NADPHox-deficient mice (Fig. 3a), which suggests that NADPHox plays a major role in the pathogenesis of OXPHOS dysfunction. Although little information exists on the effects of NADPHox on OXPHOS function, NOX4, a member of the NADPHox family located in the mitochondrial inner membrane, has been shown to inhibit complex I activity^34^. However, NOX4 cannot be responsible for the OXPHOS dysfunction found in HFD-fed mice as NOX4 was present in NOX2^−/−^ mice and OXPHOS dysfunction was not limited to complex I in these mice. Our study clearly shows that HFD markedly reduced the amount of fully assembled OXPHOS complexes in wild-type mice but not in NOX2^−/−^ mice fed the same diet (Fig. 4a). These differences may be ascribed to the absence of increased oxidative damage to mtDNA in the latter group of mice (Fig. 5c) and consequently to normal gene expression for mtDNA-encoded subunits (Fig. 5b). In addition, 3-tyrosine-nitrated mitochondrial proteins were significantly lower in NOX2^−/−^/HFD mice than in WT/HFD mice (Fig. 5e). As we and others have already shown^29^,^30^, 3-tyrosine nitration causes OXPHOS protein degradation.

The reduced gene expression of mtDNA-encoded subunits in WT/HFD mice was not due to the absence of the transcriptional factors involved in the regulation of genes encoding OXPHOS proteins, since gene expression for such regulatory factors was increased in WT/HFD mice, likely as a compensatory response to OXPHOS dysfunction. A large number of such transcription factors have been identified, including Sp1, ERRα, and PGC1α, among others^35^. In particular, PGC1α plays a critical role in the adaptation mechanisms to ATP reduction^36^. As indicated, our study shows that the gene expression of these transcriptional factors was increased only in WT/HFD mice. Indeed, this group of mice was the only one where hepatic ATP content was decreased (Fig. 3b). The cellular ATP pool plays a role in the transcription regulation of nDNA- and mtDNA-encoded subunits^37^,^38^.

Although we cannot excluded that other oxidative systems may contribute to the nitrooxidative stress present in the liver of mice fed a HFD, our results indicate that the NADPHox enzyme complex plays a key role in the pathogenesis of NASH and OXPHOS dysfunction. As the latter is also an important cellular source of ROS^39^, it may well create a vicious cycle contributing to increase oxidative stress.

In the liver of NOX2^−/−^/HFD mice, the amount of both UCP-2 protein, which may help to decrease ATP and ROS formation^40^, and PPARγ, a transcription factor that upregulates *Ucp-2* gene expression, was significantly lower than in WT/HFD mice and remained at about the same levels seen in WT/SCD mice. Similarly, the silencing of NADPHox abrogated the upregulation of UCP2 and PPARγ protein expression caused by saturated fatty acids in HepG2 cells (Supplementary Fig. S4). In previous studies, we showed that these acids increased NADPHox activity and caused oxidative and nitrosative stress^16^. This behaviour of UCP-2 in NOX2^−/−^/HFD mice and HepG2 cells with silenced NADPHox may be ascribed to the fact that UCP2 protects cells against oxidative stress^40^. Therefore, the increase in UCP2 and PPARγ in WT/HFD mice and HepG2 cells treated with saturated fatty acids should be interpreted as a response to the oxidative stress caused by increased NADPHox activity in these animals and cells.

Our study suggests that the NADPHox complex may play a key role in the pathogenesis of changes caused by HFD in the mouse liver. The mechanisms by which HFD elevates this enzymatic activity were not investigated in this study; however, it has been shown that saturated fatty acids^16^,^41^,^42^, TGFβ1^43^, and TNFα^44^, all of which are increased in the liver of HFD-fed mice (Table 1; Fig. 2a), can elevate NAFDPHox activity. Saturated fatty acids, but not the monounsaturated oleic acid, may increase NADPHox activity by upregulating NADPHox component gene expression and by enhancing p47^*phox*^ phosphorylation^16^. TGFβ1 induces the gene expression of NOX4, a member of the NADPHox gene family, acting on an AP1/Smad binding box in the NOX4 gene promoter^45^; also TNFα, in addition to inducing oxidative stress by disrupting the mitochondrial electron transport chain^46^, may increase NADPHox activity by activating NFkB^44^.

In conclusion, our study clearly shows that most hepatic histological changes, OXPHOS dysfunction, reduced OXPHOS subunits, and some features of the metabolic syndrome caused by HFD in wild-type mice are not present in NADPHox-deficient mice fed the same diet, which suggests that this oxidative system plays a critical role in the pathogenesis of NASH and in the mechanisms responsible for OXPHOS dysfunction.

## Methods

### Animal model of NAFLD

All procedures were carried out in accordance with the Spanish Guidelines for the Care and Use of Laboratory Animals. All the experimental protocols involved were approved by the “Ethic and Animal Welfare Commission of University Hospital “12 de Octubre” Madrid, Spain. The three-week-old C57BL/6J wild-type and NADPHox-deficient (NOX2^−/−^) mice were purchased from Charles River Laboratory (Charles River Laboratories España, SA. Santa Perpetua de la Mogoda. Spain). Animals were housed at constant room temperature (23 °C) under 12 hour light/dark cycles with *ad libitum* access to water and laboratory diet. Twenty-four C57BL/6J mice were distributed in four groups: (1) Group WT/SCD included six wild-type mice fed a standard chow diet (SCD) (5LF2 EURodent Diet 14%. LabDiet, St Louis, MO); (2) Group WT/HFD contained six wild-type mice on a HFD (Harlan Laboratories, Madison, WI) consisting of 21.2% (42% kcal) fat, 17.3% (15.2% kcal) protein, and 35% (42.7% kcal) carbohydrate; (3) Group NOX2^−/−^*/*SCD was composed of six NOX2-deficient mice (Supplementary Fig. S5) fed a standard chow diet; and (4) group NOX2^−/−^/HFD included six NOX2-deficient mice on a HFD. Diets were maintained for 32 weeks. Body weight and food intake were measured every two weeks. Food intake was obtained by subtracting the remaining food, including any spilled food in the cage, from a weighed aliquot for a period of one week. Caloric intake was calculated on the basis of 3.18 kcal/g for SCD and 4.5 kcal/g for HFD. Food, but not water, was withdrawn overnight before sacrifice. Animals were anesthetised and sacrificed at 35 weeks of age, and their livers were rapidly harvested for further analysis. A portion of liver tissue was placed in a 10% formaldehyde solution and routinely processed for histological assessment. Sections were stained with hematoxylin-eosin, and with Masson trichrome. Protein nitration by peroxynitrite [3-nitrotyrosine (3NT)] in the liver was assessed as described elsewhere^6^. Plasma glucose, triglyceride, and aminotransferase levels were measured using a conventional automatic analyser. Plasma FFA levels were determined using the “Free Fatty Acids, Half Micro Test” kit (Roche Diagnostics GmbH, Penzberg, Germany). Triglyceride concentration in liver tissue was measured using a serum triglyceride determination kit (Sigma-Aldrich Química SA. Tres Cantos. Spain) following the manufacturer’s indications.

### Cell culture

The HepG2 cell line obtained from American Type Culture Collection (Manassas, VA) was grown at 37 °C in an atmosphere of 5% CO2, 95% air in cell culture flask using 10 ml of Dulbecco’s Modified Eagle’s Medium (Lonza Iberica SA, Barcelona, Spain) containing 10% fetal calf serum, 1% L-glutamine, 1% penicillin, 1% streptomycin, 1% Fungizone. Cells were plated at a density of 5 × 106/80-cm^2^ flask. The effect of fatty acids was examined by adding these agents to the cells cultured in medium with 2% fetal calf serum. Palmitic and stearic fatty acids were dissolved as described by Joshi-Barve *et al*.^47^.

### OXPHOS enzyme activity assays

The enzyme activity of OXPHOS complexes was measured in frozen liver tissue as described elsewhere^6^, expressed as nanomoles of substrate used per minute per milligram of protein, and, to correct for the hepatic content of mitochondria, referred as percentage of specific citrate synthase (CS) activity.

### Assessment of full assembly of OXPHOS complexes

OXPHOS complexes were isolated by one-dimensional BN-PAGE as described elsewhere^8^. Following electrophoresis, proteins were transferred to a polyvinyl difluoride membrane (0.45-μm pore size) (Immobilon-P transfer Membrane; Millipore Co., Bedford, MA, USA). Western blotting for these proteins was performed using primary antibodies against complex I subunits NDUFA9, complex II subunit SDHA, complex III subunit UQCRC2, complex IV subunit COX4, complex V subunit ATP5A1 (Molecular Probes Inc., Eugene, OR, USA), and TOM20 (loading control) (Santa Cruz Biotechnology, Inc. Santa Cruz, CA) in blocking buffer for 2 h. After washing, blots were incubated for 1 h with peroxidase-conjugated antibody as secondary antibody, prepared at a 1:5,000 dilution (Molecular Probes Inc.). Immunoreactive material was visualized by chemiluminescence (ECL, Western Blotting Detection; GE Healthcare, Madrid, Spain) according to the manufacturer’s instructions. The blot was finally exposed to Hyperfilm MP (Amersham, GE Healthcare). Enhanced chemiluminescence (ECL) signals were quantified using the ImageJ image analysis software^48^.

### Second dimension electrophoresis for the assessment of complex subunits

For second dimension BN/SDS-PAGE we followed the procedure described elsewhere^8^. Western blotting was performed using primary antibodies against subunits NDUFA9, NDUFA6, NDUFB6, NDUFS3, NDUFV1, NDUFV2, MTND1, MTND4L, and MTND6 (complex I); SDHA (complex II); UQCRC2, UQCRFS1, and MTCYTB (complex III); COX4 and MTCO1 (complex IV); ATP5A1 and MTATP8 (ATP synthase) (Molecular Probes Inc. Eugene. OR.), and VDAC1 (loading control). Antibodies against MTND1, MTND6, MTND4L, and VDAC1 were obtained from Santa Cruz Biotechnology, Inc. (Santa Cruz, CA).

Lipid peroxidation was determined by measuring thiobarbituric acid reactive substances (TBARS) in cells as described by Ohkawa *et al*.^49^. Mitochondrial reduced glutathione (GSH) was measured using the procedure described by Eady *et al*.^50^.

### Measurement of 8-OHdG in nuclear and mitochondrial DNA

Oxidative damage to nDNA and mtDNA was determined following the procedure described elsewhere^29^.

### Western blot

Proteins were separated and transferred to an Immobilon membrane (Millipore, Bedford, MA) as previously described^51^. After electrotransfer, the filters were incubated with appropriate polyclonal antibody against 3-nitrotyrosine (Upstate Biotechnology. Lake Placid. NY), iNOS, phosphorylated Rac1, NOX2, UCP2, CHOP, PPARγ, 3-nitrotyrosine (Santa Cruz Biotechnology, Santa Cruz, CA), and β-actin (Sigma-Aldrich, Alcobendas, Spain). Signals were detected using the ECL Western Blotting Detection Reagent (Amersham Ibérica, Madrid, Spain).

### Adiponectin

Adiponectin level was determined in the mouse plasma using an ELISA assay (Invitrogen, Life Technology, Frederick, MD).

### Hepatic concentrations of TNFα

This was measured using a high-sensitivity enzyme-linked immunosorbent assay according to the manufacturer’s instructions (Mouse TNF-α ELISA Kit, Life Technology Corp. Frederick, MD).

### Quantitative real-time polymerase chain reaction

Total RNA was extracted from liver tissue using the TRI-Reagent (Sigma-Aldrich, Steinheim, Germany) according to the manufacturer’s instructions. RNA was treated with DNase I to remove DNA contamination (Sigma-Aldrich, Steinheim, Germany). cDNA was generated from a 1-μg sample of RNA using the First Strand cDNA Synthesis Kit for RT-PCR (Roche Applied Science, Indianapolis, IN) at 25 °C for 5 min; 42 °C for 60 min; 95 °C for 5 min, and 4 °C for 5 min. Quantitative real-time PCR was performed as described elsewhere^52^. Amplification conditions included 45 cycles of denaturation at 95 °C for 10 sec, annealing at 59 °C for 5 sec, and extension at 72 °C for 20 sec^53^. The correct size and purity of amplified products was verified by agarose gel electrophoresis. Sequence of primers used in these experiments are shown in Supplementary Table S1.

### Measurement of total ATP content and ATP/ADP ratio in the mouse liver

Liver samples were homogenized in perchloric acid and centrifuged at 15,000 G for 2 minutes. Supernatants were collected and 30 μl were added to a 96-well plate and then brought up to 50 μl with ATP assay buffer. The ATP reaction mix and ATP measurement was performed using the ATP Colorimetric/Fluorometric Assay Kit (BioVision Research Products, Milpitas, CA) according to the manufacturer’s protocol. The ADP/ATP ratio was measured by luminometry using the commercial assay kit ApoSENSOR^TM^ ADP/ATP Ratio Assay Kit (BioVision Research Products, Mountain View, CA).

NADPHox activity was measured following the procedure described by Jalil *et al*.^54^.

### Statistical analysis

These analyses were carried out using the SPSS Statistical Software for Windows, version 9 (SPSS Inc., Chicago, IL, USA). The unpaired ***t***-test was used to assess the significance of differences between means. All results were expressed as mean ± SD. P-values < 0.05 were considered significant.

## Additional Information

**How to cite this article**: García-Ruiz, I. *et al*. NADPH oxidase is implicated in the pathogenesis of oxidative phosphorylation dysfunction in mice fed a high-fat diet. *Sci. Rep.* **6**, 23664; doi: 10.1038/srep23664 (2016).

## Supplementary Material

Supplementary Information

## Figures and Tables

**Table 1:** Characteristics and laboratory features of the four groups of mice.

**Characteristics/features**	**WT/SCD (n, 6)**	**WT/HFD (n, 6)**	**NOX**^−/−^**/SCD (n, 6)**	**NOX**^−/−^**/HFD (n, 6)**
Initial weight (g) (Week, 3)	12.6 ± 1.2	12.3 ± 1.3 (ns)	11.40 ± 1.04 [NS]	11.70 ± 0.98 (ns) [ns]
Final weight (g) (Week, 35)	32.8 ± 1.8	49.9 ± 1.2***	21.2 ± 1.4 [C]	41.3 ± 4.8*** [c]
Weight gain (g)	20.2 ± 3.4	37.4 ± 2.9***	9.9 ± 1.5 [C]	30.5 ± 3.7*** [b]
Weight gain [%]	160.3 ± 20.5	304.1 ± 24.6***	89.8 ± 10.1 [C]	260.6 ± 11.1*** [b]
Caloric intake (Kcal/day)	11.4 ± 1.2	13.7 ± 1.1**	11.8 ± 1.2 [NS]	14.4 ± 1.2** [ns]
Liver weight (g)	1.70 ± 0.14	4.23 ± 0.22***	1.32 ± 0.26 [NS]	2.89 ± 0.14*** [ns]
Liver weight/body weight (%)	5.18 ± 0.31	8.47 ± 0.17***	6.20 ± 0.59 [B]	7,02 ± 0.34* [c]
Hepatic triglycerides (mg/g liver tissue)	7.6 ± 2.4	37.0 ± 2.6***	9.2 ± 1.3 [NS]	15.05 ± 3.9** [c]
Hepatic FFAs (mmol/g protein)	8.1 ± 0.4	28.5 ± 1.1***	9.4 ± 0.6 [C]	10.7 ± 1.5 (ns) [c ]
Hepatic TNFα (pg/mg protein)	2.3 ± 0.3	30.0 ± 5.1***	3.8 ± 1.3 [A]	5.1 ± 2.8 (ns) [c]
Plasma glucose (mg/dL)	121.2 ± 11.4	221.5 ± 35.2***	139.0 ± 16.0 [NS]	148.0 ± 13.0 (ns) [c]
Plasma triglycerides(mg/dL)	78.6 ± 21.6	177.1 ± 29.0***	65.0 ± 5.5 [NS]	75.9 ± 19.1 (ns) [c]
Plasma free fatty acids (mmol/L)	0.23 ± 0.03	0.61 ± 0.04***	0.20 ± 0.03 [NS]	0.23 ± 0.03 (ns) [c]
Plasma adiponectin (µg/mL)	15.1 ± 3.2	7.4 ± 1.3***	15.0 ± 1.7 [NS]	13. 9 ± 1.2 (ns) [c]
Plasma AST(IU/L)	44.5 ± 13.3	181.0 ± 21.5***	47.7 ± 23.5 [NS]	79.6 ± 32.6 (ns) [c]
Plasma ALT (IU/L)	14.3 ± 9.9	328.7 ± 48.2***	10.5 ± 2.2 [NS]	67.2 ± 49.2* [c]

WT/SCD, wild-type mice on a standard chow diet; WT/HFD, wild-type mice on a HFD; NOX^−/−^/SCD, NADPH oxidase-deficient mice on a standard chow diet; NOX^−/−^/HFD, NADPH oxidase-deficient mice on a HFD; % weight gain is shown as percentage of initial weight. (ns), not significant; *p < 0.05; **p < 0.01; ***p < 0.001 as compared mice fed a HFD with mice on a SCD. [NS], not significant; [A], p < 0.05; [B], p < 0.01; [C], p < 0.001 as compared NOX2^−/−^/SCD mice with WT/SCD mice. [ns], not significant; [b], p < 0.01; [c], p < 0.001 as compared NOX2^−/−^/HFD mice with WT/HFD mice.
