# Supplementary material for: NADPH oxidase is implicated in the pathogenesis of oxidative phosphorylation dysfunction in mice fed a high-fat diet
Source: Sci Rep. 2016 May 13;6:23664. doi: 10.1038/srep23664 (PMC4866080; doi:10.1038/srep23664)
Supplement: Supplementary Information [file srep23664-s1.pdf]

NADPH oxidase is implicated in the pathogenesis of oxidative phosphorylation dysfunction in mice fed a high-fat diet

Inmaculada García-Ruiz [1 *], Pablo Solís-Muñoz [2], Daniel Fernández-Moreira [3], Montserrat Grau [1], Teresa Muñoz-Yagüe [1], José A. Solís-Herruzo [1]

Supplementary material

Figure legends.

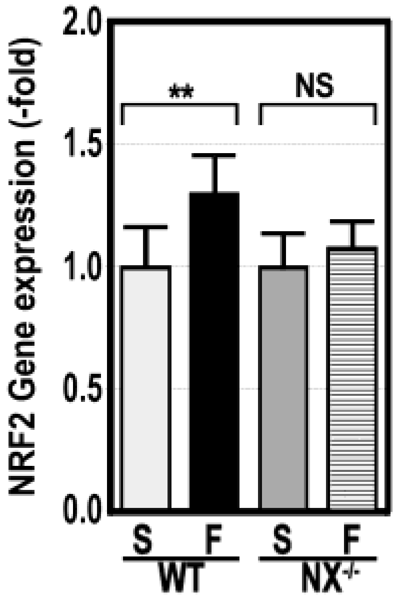

Supplementary Figure S1. Nrf2 gene expression is significantly increased in mice on a HFD. The gene expression of Nrf2 was measured by RT-PCR. **WT**, wild-type mice; **NX**$^{-/-}$, NADPH oxidase-deficient mice; **S**, mice on a standard chow diet; **F**, mice on a high-fat diet. **, $p<0.01$; NS, not significant.

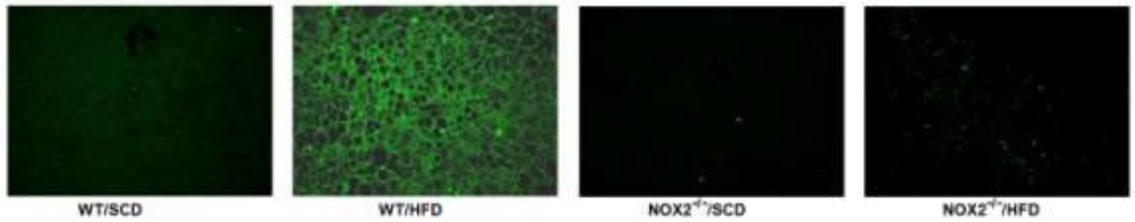

Supplementary Figure S2. Hepatic proteins are 3-tyrosine-nitrated in WT mice on a HFD but not in NADPHox-deficient mice on the same diet. Wild-type (WT) and NADPHox ($NOX2^{-/-}$)-deficient mice were fed either a standard chow diet (SCD) or a high-fat diet (HFD). Formalin-fixed, paraffin-embedded sections were deparaffinized and then incubated with anti-3-nitrotyrosine antibody, followed by incubation with anti-rabbit IgG FITC conjugated secondary antibody.

.

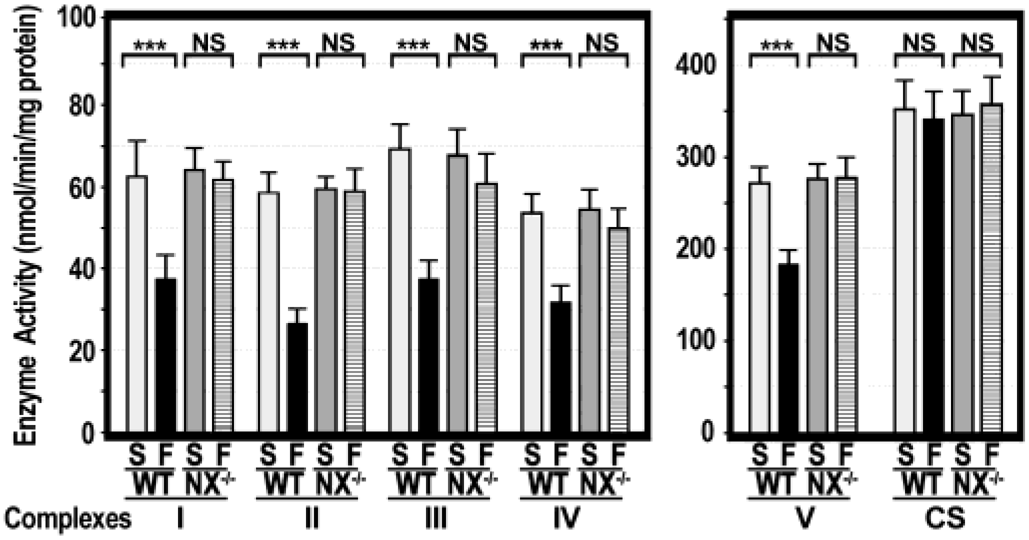

Supplementary Figure S3. The enzyme activity of OXPHOS complexes is decreased in the visceral fat of obese wild-type mice (WT) but not in the visceral fat of obese NADPHox-deficient mice ($NX^{-/-}$). The activity of the OXPHOS enzyme complexes and citrate synthase (CS) was measured in fat of the greater omentum of the four groups of mice and expressed as nmol of substrate used per minute per mg protein and was referred to as a percentage of the specific activity of citrate synthase (CS). Values are shown as mean ± SD. NS, not significant; ***, $p<0.001$; S, mice fed a standard chow diet; F, mice fed a high fat diet.

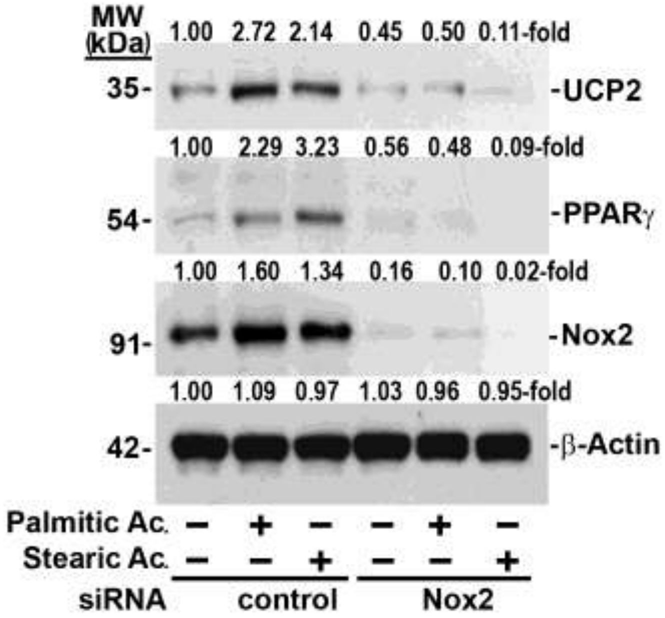

Supplementary Figure S4. The silencing of *NOX2* gene expression prevents the increased protein expression of UCP2, PPARγ, and NOX2 that results from treating HepG2 cells with saturated fatty acids. UCP2, PPARγ, NOX2, and β-actin protein expression was measured in confluent HepG2 cells treated either with control medium or with 200 μM of palmitic or stearic acid for 24 hours.

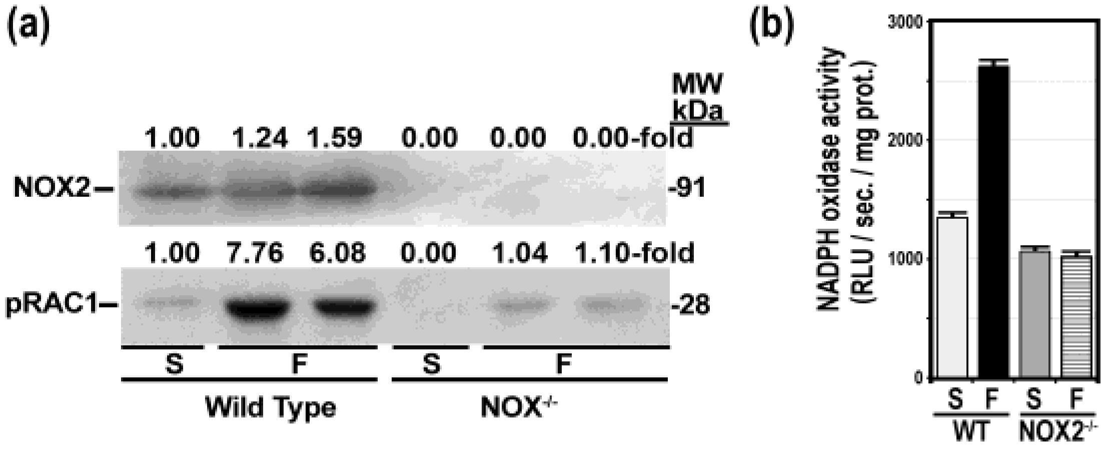

Supplementary Figure S5. NOX2 protein expression is absent, and phosphorylated Rac1 and NADPH oxidase activity is reduced in NADPH-deficient mice. (a) Western blot showing NOX2 and phosphorylated RAC1 protein expression in the four groups of mice. (b) NADPH oxidase activity in the four groups of mice. WT, wild-type mice; NOX2$^{-/-}$, NOX2-deficient mice; S, mice fed a standard chow diet; F, mice fed a high fat diet. ***, $p<0.001$.

Supplementary Table S1.

Primers used in quantitative real-time polymerase chain reaction

Primer name	Direction	Sequence
**Mouse NDUFA9**	Sense<br>Antisense	5′-CAT TAC TGC AGA GCC ACT-3′<br>5′-ATC AGA CGA AGG TGC ATG AT-3′
**Mouse NDUFB6**	Sense<br>Antisense	5′-ATA ACT TTT TGC GGG ACG GG-3′<br>5′-CAG GAA AAT CTC TCA TTG GTG-3′
**Mouse NDUFS3**	Sense<br>Antisense	5′-AGG AAC ATG GCG GCG GCT GC-3′<br>5′-ATT TCA GCC ACA TAC TCT CC-3′
**Mouse MTND1 (ND1)**	Sense<br>Antisense	5′-TGC ACC TAC CCT ATC ACT C-3′<br>5′-ATT GTT TGG GCT ACG GCT C-3′
**Mouse MTND2 (ND2)**	Sense<br>Antisense	5′-ATG AGT AGG CCT GGA ATT C-3′<br>5′-ATC AGA AGT GGA ATG GGG C-3′
**Mouse MTND4 (ND4)**	Sense<br>Antisense	5′-ATA ATT ATA ACT AGC TCA ATC TGC-3′<br>5′-TCG TAG TTG GAG TTT GCT AG-3
**Mouse MTND4L (ND4L)**	Sense<br>Antisense	5′-CTC ACC ATA GCC TTC TCA C-3′<br>5′-CGT AAT CTG TTC CGT ACG TG-3′
**Mouse MTND6**	Sense<br>Antisense	5′-TGT ATG AGG TTG ATG ATG TTG G-3′<br>5′-CCG CAA ACA AAG ATC ACC C-3′
**Mouse UQCRC1 (core 1)**	Sense<br>Antisense	5′-CCT ACA GCA CTC GAG AGC AC-3′<br>5′-AGG TGT GCC CTG GAA TGC TG-3′
**Mouse UQCRC2 (core 2)**	Sense<br>Antisense	5′-TCC CTC AAA GTT GCC CC-3′<br>5′-GCA AGA CGT AGT AAA TGT GAG-3′
**Mouse UQCRFS (FeS)**	Sense<br>Antisense	5′-GAT GTC AAG GTG CCC GAC TT-3′<br>5′-GAT CTC GAT CTT CGA CAT GG-3′
**Mouse MTCYB**	Sense<br>Antisense	5′-CTT TGG GTC CCT TCT AGG AGT CTG-3′<br>5′-GCT GTG GCT ATG ACT GCG AAC AG-3′
**Mouse SDHA (70 kDa)**	Sense<br>Antisense	5′-CAT ACT GTT GCA GCA GCA CAG G-3′<br>5′-CCA CCA AAT GCA CGC TGA TA-3′
**Mouse COX4 (COX IV)**	Sense<br>Antisense	5′-GAG CAC ATG GGA GTG TTG TG-3′<br>5′-CTG TCT TCC ATT CAT TGG TGC C-3′
**Mouse MTCOX1**	Sense<br>Antisense	5′-GCT GAA GGA GAA GGA GAA G-3′<br>5′-ATA CAC ATA GCT CTT CTC CC-3′
**Mouse ATP5**	Sense<br>Antisense	5′-GGT CAT CCT TTG TTG GTG C-3′<br>5′-GAG AAT TCC ACC ATC CCT TC-3′
**Mouse MTATP6**	Sense<br>Antisense	5′-CCA CAC ACC AAA AGG ACG AAC ATG A-3′<br>5′-CGG ACT GCT AAT GCC ATT GGT TG-3′
**Mouse TNFα**	Sense	5′-GAG GCA CTC CCC CAA AAG-3′

	Antisense	5′-GGG TCT GGG CCA TAG AAC TG-3′
**Mouse IFNγ**	Sense<br>Antisense	5′-CTC AAG TGG CAT AGA TGT GG-3′<br>5′-CAG GTG TGA TTC AAT GAC GC-3′
**Mouse MCP-1**	Sense<br>Antisense	5′-CCA CGT GTT GGC TCA GC-3′<br>5′-AGC ACA GAC CTC TCT CTT G-3′
**Mouse CRP**	Sense<br>Antisense	5′-GAC ACT CAG CCC CAA TGT TTT G-3′<br>5′-GTC CTC TAG TGC TGA GGA C-3′
**Mouse Caspase 3**	Sense<br>Antisense	5′-GGA TGT GGA CGC AGC CAA-3′<br>5′-CCT TCA TCA CCA TGG CTT AG-3′
**Mouse Collagen 1α(I)**	Sense<br>Antisense	5′-CTT CCC TGG TGC AGT TGG T-3′<br>5′-GCA ATA CCA GGA GCA CCA TTG-3′
**Mouse TGFβ**	Sense<br>Antisense	5′-CAG AGC TGC GCT TGC AGA GAT TAA-3′<br>5′-CAG TGA GCG CTG AAT CGA AAG C-3′
**Mouse NOX2**	Sense<br>Antisense	5′-GCA GCC TGC CTG AAT TTC AAC T-3′<br>5′-AGA GAG AGC TAT TGA ATA CCG G-3′
**Mouse PGC1α.**	Sense:<br>Antisense:	5′-GAG ACT TTG GAG GCC AGC AAG-3′<br>5′-CCA AGG GTA GCT CAG TTT ATC AG-3′
**Mouse ERRα**	Sense:<br>Antisense:	5′-GAC AGT CCA AAG GGT TCC TCA G-3′<br>5′-CTG GAT GGT CCT CTT GAA GAA GGC-3′
**Mouse SP1**	Sense:<br>Antisense:	5′-CTG CCA GCT TGG TGT CAT CAC-3′<br>5′-CTG ACT TCC TTG CAG CGA GC-3′
**Mouse αSMA**	Sense:<br>Antisense:	5′- CCC AGA CAT CAG GGA GTA ATG G-3′<br>5′- TCT ATC GGA TAC TTC AGC GTC A-3′
**Mouse NRF2**	Sense:<br>Antisense:	5′-CTG GAT GAA GAA ACA GGA GAA TTC C-3′<br>5′-CTC TGC CAA AAG CTG CAT ACA GTC-3′
**Mouse β-Actin**	Sense<br>Antisense	5′-ATG GAT GAC GAT ATC GCT G-3′<br>5′-GTT GGT AAC AAT GCC ATG TTC-3′
